# Increased dipeptidyl peptidase 4 in patients with concomitant transthyretin cardiac amyloidosis and severe aortic stenosis

**DOI:** 10.1016/j.ijcha.2025.101820

**Published:** 2025-10-08

**Authors:** Margrethe Flesvig Holt, Annika E. Michelsen, August Flø, Kristoffer Russell, Jan Otto Beitnes, Sophie Foss Kløve, Anders Hodt, Lars Gullestad, Pål Aukrust, Einar Gude, Kaspar Broch, Thor Ueland

**Affiliations:** a Department of Cardiology, Oslo University Hospital, Rikshospitalet, Oslo, Norway; b Research Institute of Internal Medicine, Oslo University Hospital, Rikshospitalet, Oslo, Norway; c Institute of Clinical Medicine, Faculty of Medicine, University of Oslo, Norway; d Department of Nuclear Medicine, Oslo University Hospital, Oslo, Norway; e Section of Clinical Immunology and Infectious Diseases, Oslo University Hospital Rikshospitalet, Oslo, Norway; f Thrombosis Research Center (TREC), Division of Internal Medicine, University Hospital of North Norway, Tromsø, Norway

**Keywords:** Aortic stenosis, Transthyretin amyloid cardiomyopathy, Decorin, Dipeptidyl peptidase 4, Hepatocyte growth factor

## Abstract

**Background:**

Due to overlapping symptoms and signs, it can be challenging to diagnose transthyretin amyloid cardiomyopathy (ATTR-CM) in the setting of concomitant aortic stenosis. Biomarkers may discriminate between heart failure with ATTR-CM and heart failure without ATTR-CM, but it is not known if these markers can differentiate between AS with and AS without concomitant ATTR-CM.

**Methods:**

In 9 patients with ATTR-CM and AS, 161 patients with lone AS, and 23 healthy controls, we measured 8 plasma proteins previously identified by proteomic analysis as potential candidates for diagnosing ATTR-CM. We assessed differences between groups and association with indices of heart failure and AS severity.

**Results:**

Plasma levels of dipeptidyl peptidase 4 (DPP4) were significantly higher in patients with AS and ATTR-CM than in patients with lone AS and in healthy controls. Lower levels of DPP4 were also associated with worse left ventricular function, higher New York Heart Association functional class, and low-flow, low-gradient aortic stenosis.

**Conclusions:**

Our findings suggest that DPP4 may be a marker of ATTR-CM in patients with severe AS. In all AS patients, those with and without coexisting ATTR-CM, high DPP4 levels were asociated with better cardiac function.

## 1 Introduction

Transthyretin cardiomyopathy (ATTR-CM) is an infiltrative cardiomyopathy caused by the deposition and aggregation of amyloid fibrils in the extracellular space [1]. It is an increasingly recognized cause of heart failure (HF) in the elderly. The prevalence of aortic stenosis (AS) also increases with age, and AS is the most common valvular disease in the elderly [2]. ATTR-CM and AS share several epidemiological, clinical, echocardiographic, and biochemical features [2]. The challenge lies in identifying ATTR-CM in the context of coexisting AS. Differentiating between the two conditions can be difficult due to their overlapping characteristics. The reported prevalence of ATTR-CM in patients with AS ranges from 4 – 16 % [2]. The European Society of Cardiology (ESC) recommends screening for ATTR-CM in patients ≥ 65 years of age with ventricular hypertrophy and AS [1]. However, screening all patients with AS for ATTR-CM using bone scintigraphy and assessment of monoclonal protein is resource demanding and potentially low-yield.

Using targeted proteomic analyses, we have recently identified candidate proteins for discriminating between ATTR-CM and other causes of HF [3]. In this study, we evaluated if plasma levels of these markers are differentially regulated in patients with AS patients with ATTR-CM vs patients with AS without ATTR-CM. We also assessed their associations with cardiac function and AS severity.

## 2 Methods

We used bone scintigraphy to screen patients with severe AS who were accepted for transcatheter aortic valve implantation (TAVI), for ATTR-CM. As previously described, we defined ATTR-CM as a cardiac tracer uptake ≥ Perugini grade 2 in the absence of abnormal monoclonal protein and abnormal free light-chain-ratio in serum [4]. Endomyocardial biopsy was performed if there were signs of light-chain disease or ambiguous bone scans. Aortic stenosis was assessed in accordance with the 2021 ESC/EACTS guidelines [5]. Strain analyses were performed irrespective of heart rhythm. Global longitudinal strain (GLS) was reported as negative values, with high strain values (low absolutes) indicating poor left ventricular function. Venous blood samples for immunoassays were collected in EDTA tubes prior to TAVI, immersed in melting ice, and centrifuged at 2,100 g at 4 °C for 20 min to obtain platelet-poor plasma. All samples were stored at −80 °C.

Levels of the TNF Receptor Superfamily Member 13B (TNFSF13B), C-X-C motif chemokine ligand 9 (CXCL9) (PeproTech®, Rocky Hill, NJ), Dipeptidyl peptidase 4 (DPP4), Hepatocyte growth factor (HGF), and Galectin-9 (Gal-9), transforming growth factor beta receptor 3 (TGFβR3), and decorin (all from R&D Systems, Minneapolis, MN) and Apolipoprotein M (APOM) (MabTech, Nacka Strand, Sweden) were quantified by Enzyme Immunoassays, with intra- and inter-assay coefficients of variation < 10 %. The Regional Committee for Medical Research Ethics and the local Data Protection Officer at Oslo University Hospital approved the study. All patients provided written informed consent before inclusion.

Statistics were performed using SPSS version 29.0.0.0. (IBM, Armonk, NY, USA). Baseline characteristics are expressed as mean ± standard deviation, median (interquartile range), or numbers (percentage), as appropriate. We used Chi-square tests, one-way analysis of variance (ANOVA) or Kruskal-Wallis tests to assess differences between groups. The ability of each protein to differentiate lone AS from AS and ATTR-CM was assessed by receiver operating characteristic (ROC) analyses. To compare protein levels between groups, we used multivariate general linear models (GLM) with proteins as target, diagnostic group (or dichotomized indices of HF and AS severity) as fixed factor, and age and sex as covariates. We also performed multivariable logistic regression to assess the association between DPP4 and concomitant AS and ATTR-CM. For adjustment we used age as well as a propensity score (PS1) made from age, troponin T (cTnT), N-terminal pro-B-type natriuretic peptides (NT-proBNP), C-reactive protein (CRP), eGFR and use of DPP4 inhibitors. This PS-score was also generated with DPP4 (PS2) enabling comparison (i.e. PS1 vs PS2) in paired ROC analysis. Correlations between protein levels and measures of cardiac disease and function were assessed by Spearman’s rho. P-values are two-sided.

## 3 Results

**Fig. 1:**
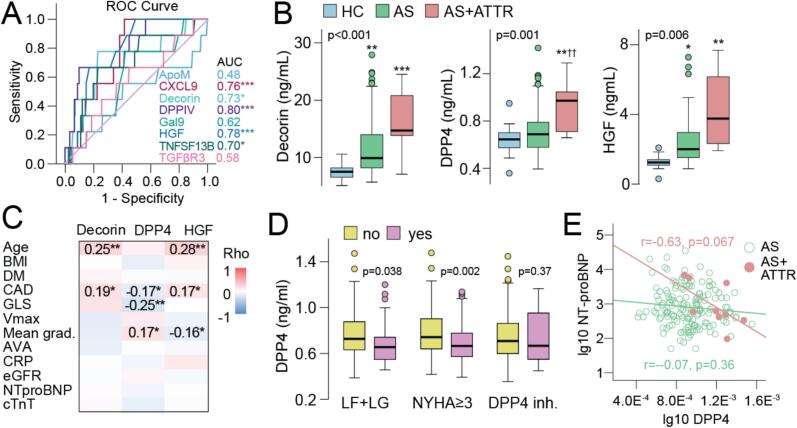
**Dysregulated proteins in patients with lone aortic stenosis (AS) and concomitant aortic stenosis and transthyretin amyloid cardiomyopathy (AS + ATTR). A)** ROC analysis of proteins in relation to presence of ATTR-CM (n = 9) in the study population. *p < 0.05, ***p < 0.001. **B)** Tukey plots showing dysregulated proteins in AS with (n = 9) and without (n = 161) ATTR-CM in relation to healthy controls (HC) (n = 23). P values reflect the group effect from multivariate general linear model with age and sex as covariates. *p < 0.05, **p < 0.01, ***p < 0.001 vs*.* HC; ^††^p < 0.01 vs*.* AS. **C)** Heatmap showing correlations (Spearman) between plasma proteins and echocardiographic, hemodynamic and biochemical indices of cardiac function. *p < 0.05, **p < 0.01. **D)** Tukey plots showing Dipeptidyl Peptidase 4 (DPP4) levels in aortic stenosis patients with and without low-flow low-gradient aortic stenosis (LF + LG) (left), in New York Heart Association functional class (NYHA) ≥ 3 and < 3 (middle), and according to use of DPP4 inhibitors (n = 9 using DPP4 inhibitors). **E)** Correlation plots showing associations between DPP4 and N-terminal pro-brain natriuretic peptide (NT-proBNP) in patients with AS with (red) and without (green) concomitant ATTR-CM. AUC, Area under the curve; ApoM, Apolipoprotein M; CXCL9, C-X-C Motif Chemokine Ligand 9; Gal9, Galectin-9; HGF, Hepatocyte Growth Factor; TNFSF13B, TNF Receptor Superfamily Member 13B; TGFβR3, Transforming Growth Factor Beta Receptor 3; BMI, body mass index; DM, diabetes mellitus; CAD, coronary artery disease; Vmax, Aortic valve peak velocity; Mean grad., Mean aortic pressure gradient; AVA, Aortic valve peak velocity; CRP, C-reactive protein; eGFR estimated glomerular filtration rate; cTnT, Cardiac troponin T. (For interpretation of the references to colour in this figure legend, the reader is referred to the web version of this article.)

Patient characteristics are listed in Table 1. The healthy controls were on average younger than the patients. Patients with AS and ATTR-CM were older than patients with lone AS, more often used beta-blockers, and more often had low-flow, low-gradient AS. CXCL9, HGF, and DPP4 each discriminated well between lone AS and concomitant AS and ATTR-CM (Fig. 1A). In analyses adjusted for age and sex, circulating levels of decorin and HGF were higher in patients with lone AS, and in particular in patients with AS and ATTR-CM, than in healthy controls (Fig. 1B). Notably, DPP4 levels were elevated in ATTR and AS, but not in lone AS, compared with healthy controls. (Fig. 1B). Levels of TNFSF13B (p = 0.64) and CXCL9 (p = 0.24) did not differ significantly between the three groups.

**Table 1:** Baseline characteristics of study groups.

	Healthy controls (n = 23)	AS (n = 161)	AS + ATTR (n = 9)
Age, years	65 ± 8	79 ± 7*	84 ± 5*
Female gender, n (%)	11 (47.8)	71 (44.1)	3 (33.3)
Body mass index, kg/m^2^	25 ± 2.8	27.4 ± 5.1	25.4 ± 2.6
NYHA class ≥ III, n (%)		69 (44.5)	3 (33.3)
Systolic blood pressure, mmHg		141 ± 27	136 ± 29
Diastolic blood pressure, mmHg		74 ± 12	66 ± 8
Coronary artery disease, n (%)	0 (0)	73 (45.3)	6 (66.7)
Type 2 diabetes, n (%)	0 (0)	40 (24.8)	0 (0)
Hypertension, n (%)	0 (0)	96 (59.6)	4 (44.4)
Atrial fibrillation, n (%)	0 (0)	48 (29.8)	2 (22.2)
Previous stroke or TIA, n (%)	0 (0)	23 (14.3)	0 (0)
ACEIs/ARBs, n (%)	0 (0)	102 (63.4)	4 (44.4)
Beta-blockers, n (%)	0 (0)	72 (44.7)	8 (88.9)^†^
Loop diuretics, n (%)	0 (0)	48 (29.8)	5 (55.6)
Antiplatelet therapy, n (%)	0 (0)	79 (49.1)	5 (55.6)
Statins, n (%)	0 (0)	107 (66.5)	4 (44.4)
DPP4 inhibitors, n (%)	0 (0)	9 (5.6)	0 (0)
LVEF < 50 %, n (%)	0 (0)	40 (31.3)	1 (12.5)
Aortic valve peak velocity, m/s		4.33 ± 0.67	3.88 ± 0.41^†^
Mean aortic pressure gradient, mmHg		49 ± 14	38 ± 9^†^
Aortic valve area, cm^2^		0.7 ± 0.2	0.7 ± 0.1
Low-flow, low-gradient aortic stenosis, n (%)	0 (0)	42 (26.6)	6 (66.7)^†^
Left ventricular hypertrophy, n (%)	0 (0)	102 (65)	7 (77.8)
Hemoglobin, g/dL	14.1 ± 1.1	13.4 ± 1.6	13.1 ± 1.4
C-reactive protein, mg/L	0.95 (0.65,1.9)	1.9 (0.8,4.4)	3.1 (1.2,4.8)
Creatinine, µmol/L	70 (64,81)	86 (72,99)	77 (69,132)
eGFR, ml/min/1.73 m^2^		69 (55,82)	71 (47,79)
cTnT, ng/L		19 (14,32)	21 (14,39)
NT-proBNP, ng/L		643 (321,1872)	513 (419,4600)

AS, aortic stenosis; AS + ATTR, concomitant aortic stenosis and transthyretin amyloid cardiomyopathy; NYHA, New York Heart Association; TIA, Transient ischemic attack; ACEI, Angiotensin-converting enzyme inhibitors; ARB, Angiotensin receptor blockers; LVEF, left ventricular ejection fraction; eGFR, estimated glomerular filtration rate; cTnT, Cardiac troponin T; NT-proBNP, N-terminal pro b-type natriuretic peptide. * p < 0.05 vs. healthy control. ^†^ p < 0.05 vs. aortic stenosis.

Traditional risk markers such as cTnT, NT-proBNP, CRP and eGFR gave poor discrimination for concomitant AS and ATTR-CM with AUCs between 0.48–0.53 (p > 0.78 for all), while age gave an AUC of 0.72 (p = 0.003) and a propensity score using age and all cardiac markers (PS1) gave an AUC of 0.74, p < 0.001. Comparing this propensity score (PS) with one including DPP4 (PS2, AUC of 0.86, p < 0.001) significantly improved prediction of AS + ATTR-CM when comparing ROC curves (p = 0.012). In logistic regression, DPP4 (expressed as log10 z-score) was associated with an OR of 3.17 (CI: 1.49–6.78, p = 0.003) for having AS and ATTR-CM. The odds ratio (OR) was slightly augmented when age (OR 3.63, 1.56–8.43, p = 0.003) or the PS-score was included in the model (OR 3.45, 1.54–7.74, p = 0.003).

In patients with AS with and without ATTR-CM, levels of decorin and HGF correlated positively with age and the presence of coronary artery disease (Fig. 1C). Hepatocyte growth factor was negatively associated with the mean aortic gradient. Conversely, higher levels of DPP4 correlated with less severe cardiac involvement as reflected by lower NYHA and, higher absolute values of GLS. Fig. 1D show DPP4 levels according to categorized cardiac indexes. Thus, patients with low-flow low-gradient AS and NYHA ≥ 3 had lower DPP4 levels. Nine patients used DPP4 inhibitors, but no difference in DPP4 levels was observed when comparing user and non-users. Although there was no significant correlation between DPP4 and NT-proBNP in the patient group as a whole (Fig. 1E), there was a trend towards a negative correlation between DPP4 and NT-proBNP in patients with AS *and* ATTR-CM (r = -0.63, p = 0.07).

## 4 Discussion

Transthyretin amyloid cardiomyopathy and AS frequently coexist. This study shows that DPP4 may differentiate between patients with AS and concomitant ATTR-CM and patients with lone AS. Plasma proteins that may differentiate between patients with ATTR-CM and patients with HF without amyloidosis have been studied by different groups [3,6,7]. However, these markers have not been evaluated in patients with AS and ATTR-CM versus lone AS. Among markers that were recently identified by proteomics to be associated with ATTR-CM, DPP4 stood out as the only marker that was elevated in AS with ATTR-CM compared to lone AS.

DPP4 is a glycoprotein expressed by several cell types. It is dysregulated in diseases such as Alzheimer’s disease and diabetes [8,9]. Increased DPP4 activity has been shown in the brains of Alzheimer patients and may promote amyloid plaque formation. In experimental models this is alleviated by DPP4 inhibitors [9]. However, it is not known if DPP4 contributes to amyloid accumulation in ATTR-CM. Furthermore, DPP4 has been shown to contribute to aortic valve calcification [10] and DPP4 inhibition was associated with lower risk of AS progression in patients with diabetes and mild to moderate AS [11]. Intriguingly, in our patients with severe AS, high DPP4 levels correlated with better LV performance as reflected by GLS and less severe AS, as reflected by low levels in patients with NYHA > 3 and low-flow low-gradient AS. High DPP4 in AS patients with ATTR-CM were associated with low NT-proBNP levels, an established marker of poor prognosis in severe AS [12]. In our population, traditional cardiac markers such as cTnT and NT-proBNP, were poor predictors of AS and ATTR-CM. Besides DPP4, age was the only predictor of having both conditions. Comparing ROC curves suggested a model including DPP4 significantly improved prediction of AS and ATTR-CM. Further, an increase in DPP4 was independently associated with a ∼ 3.5 higher risk of concomitant AS and ATTR-CM per SD change. As ATTR-CM was primarily diagnosed using bone scintigraphy we were unable to compare it with DPP4, however, compared to other easily obtainable serologic markers, DPP4 hold promise in the population.

DPP4-inhibitors are glucose-lowering drugs frequently prescribed for type 2 diabetes. We observed no difference in DPP4 levels in patients receiving DPP4 inhibitors, which is supported in the literature, where DPP4 activity and plasma DPP4 seems to be dissociated and notably, the effect of DPP4 inhibition on inflammatory markers was uncertain potentially resulting in higher rather than lower levels [13]. Some studies demonstrate that certain DPP4 inhibitors increase the risk of HF hospitalization in patients with type 2 diabetes. This is particularly clear in patients with comorbidities [14], although beneficial effects were observed in patients with HF with preserved ejection fraction and in diabetics [15]. In experimental models of myocardial infarction, DPP4 inhibitors show cardioprotective effects [16]. Furthermore, high levels of DPP4 have been associated with future major adverse cardiovascular events in diabetic patients with ST elevation myocardial infarction [17]. While DPP4 inhibition is generally beneficial in myocardial infarction, it may worsen outcomes in advanced heart failure through mechanisms such as sympathetic activation and fibrosis [18,19]. These issues remain uncertain, and the negative effect of DPP4 inhibition has been linked to specific DPP4 inhibitors, not necessarily to DPP4 inhibition *per se* [20,21].

Decorin, and HGF were increased in AS but not associated with ATTR-CM, possibly due to the low power of our study. Both decorin and HGF have previously been shown to provide diagnostic information in cardiac amyloidosis. Decorin is a small leucine-rich proteoglycan and component of the extracellular matrix [22] and has been proposed as a screening tool for ATTR-CM [7]. HGF is a pleiotropic cytokine with proposed cardioprotective effects [23]. We and others have previously demonstrated that high circulating HGF is associated with poor prognosis and mortality in patients with ATTR-CM and in patients light chain cardiac amyloidosis [3,6]. However, the roles of decorin and HGF in the pathophysiology of AS are unclear, and these proteins may exert dual roles in AS, demonstrating both protective and harmful effects [24,25].

Our study has several limitations that warrant emphasis. First, the low number of patients with concomitant AS and ATTR-CM limits the statistical power and increases the risk of Type II errors. Similar, the number of patients using DPP4 inhibitors were low and the impact on DPP4 levels should be taken with caution. Second, we enrolled the participants at a tertiary care center and all included patients underwent TAVI, which limit the generalizability of our findings. The control group was younger than the patient groups, which may confound biomarker differences despite statistical adjustment. Last, the associations between biomarkers and disease do not necessarily reflect the underlying pathophysiological processes in the aortic valve or the myocardium. In this study, significantly higher plasma levels of DPP4 were observed in patients with ATTR-CM and AS compared with lone AS. Interestingly, elevated DPP4 levels were associated with better cardiac function and AS indices. Given the potential harm of DPP4 inhibition in HF, further and larger studies are needed to evaluate the role of DPP4 in patients with ATTR-CM in association with AS.

**Author disclosures**

Dr. Gullestad has received lecture fees from AstraZeneca, Boehringer Ingelheim and Novartis, and has sat on advisory boards for AstraZeneca and Boehringer Ingelheim. Dr. Broch has received lecture fees and consulting fees from Pfizer, and has sat on advisory boards for AstraZeneca, Pharmacosmos, Boehringer Ingelheim and Pfizer. Dr. Gude has received grants and honoraria for lectures from Pfizer, Boehringer Ingelheim and Novartis, and has sat on advisory boards for Pfizer. Dr. Hodt has received lecture fees from Pfizer. All other authors declare that they have no conflicts of interest.

**Sources of funding**

The study was funded in part by a grant from The Bergesen Foundation and in part by a grant from The Blix Family Foundation to dr. Flesvig Holt.

## CRediT authorship contribution statement

**Margrethe Flesvig Holt:** Writing – review & editing, Writing – original draft, Project administration, Investigation, Funding acquisition, Data curation, Conceptualization. **Annika E. Michelsen:** Writing – review & editing, Investigation, Formal analysis, Data curation, Conceptualization. **August Flø:** Writing – review & editing, Project administration, Investigation, Data curation, Conceptualization. **Kristoffer Russell:** Writing – review & editing, Methodology. **Jan Otto Beitnes:** Writing – review & editing, Methodology. **Sophie Foss Kløve:** Writing – review & editing, Data curation, Investigation. **Anders Hodt:** Writing – review & editing, Resources, Methodology, Investigation. **Lars Gullestad:** Writing – review & editing, Supervision, Investigation, Conceptualization. **Pål Aukrust:** Writing – review & editing, Supervision, Methodology, Conceptualization. **Einar Gude:** Project administration, Resources, Writing – review & editing, Supervision, Methodology, Conceptualization, Investigation. **Kaspar Broch:** Writing – original draft, Validation, Funding acquisition, Methodology, Investigation, Conceptualization, Supervision. **Thor Ueland:** Writing – original draft, Visualization, Validation, Supervision, Software, Resources, Methodology, Investigation, Formal analysis, Data curation, Conceptualization.

## Declaration of competing interest

The authors declare that they have no known competing financial interests or personal relationships that could have appeared to influence the work reported in this paper.
